# Synthesis and Structural Characterization of Stable Branched DNA G-Quadruplexes Using the Trebler Phosphoramidite

**DOI:** 10.1002/open.201200009

**Published:** 2012-04

**Authors:** Rubén Ferreira, Margarita Alvira, Anna Aviñó, Irene Gómez-Pinto, Carlos González, Valérie Gabelica, Ramon Eritja

**Affiliations:** [a] Department of Chemical and Biomolecular Nanotechnology, Institute for Advanced Chemistry of Catalonia (IQAC-CSIC), Networking Centre on Bioengineering, Biomaterials and Nanomedicine (CIBER-BBN)Jordi Girona 18-26, 08034 Barcelona (Spain); [b] Department of Chemistry and Molecular Pharmacology, Institute for Research in Biomedicine (IRB Barcelona)Baldiri i Reixac 10, 08028 Barcelona (Spain); [c] Departmento de Química Física Biológica, Instituto de Química Física ‘Rocasolano'CSIC, Serrano 119, 28006 Madrid (Spain); [d] Department of Chemistry, University of LiègeAllée de la Chimie Building B6c, 4000 Liège (Belgium)

**Keywords:** 8-aminoguanines, branched oligonucleotides, DNA structures, G-quadruplexs, oligonucleotides

## Abstract

Guanine (G)-rich sequences can form a noncanonical four-stranded structure known as the G-quadruplex. G-quadruplex structures are interesting because of their potential biological properties and use in nanosciences. Here, we describe a method to prepare highly stable G-quadruplexes by linking four G-rich DNA strands to form a monomolecular G-quadruplex. In this method, one strand is synthesized first, and then a trebler molecule is added to simultaneously assemble the remaining three strands. This approach allows the introduction of specific modifications in only one of the strands. As a proof of concept, we prepared a quadruplex where one of the chains includes a change in polarity. A hybrid quadruplex is observed in ammonium acetate solutions, whereas in the presence of sodium or potassium, a parallel G-quadruplex structure is formed. In addition to the expected monomolecular quadruplexes, we observed the presence of dimeric G-quadruplex structures. We also applied the method to prepare G-quadruplexes containing a single 8-aminoguanine substitution and found that this single base stabilizes the G-quadruplex structure when located at an internal position.

## Introduction

Guanine (G)-rich DNA sequences are able to form a noncanonical four-stranded topology called a G-quadruplex. These structures are based on the G-tetrad, also called the G-quartet, which consists of a planar arrangement of four guanine bases associated through a cyclic array of Hoogsteen hydrogen bonds in which each G accepts and donates two hydrogen bonds. G-quadruplexes can be formed in the genome, for example in telomeres[1] but also in other key biological contexts, such as oncogenic promoter elements[2] and RNA 5′-untranslated regions (UTR) in close proximity to translation start sites.[3] Thus, quadruplex motifs could act as topological switches that might regulate gene expression. For all these reasons, structures of biological G-quadruplex DNA are a potential target for drug design.[4] Moreover, G-quadruplexes also have biological interest as therapeutic agents. Aptamers are short DNA- or RNA-based oligonucleotides selected from large combinatorial pools of nucleic acid sequences for their ability to bind to a specific protein.[5] Among several structures, the G-quadruplex motif is present in several aptamers with biological interest, such as the thrombin binding aptamer[6] and the anti-HIV-1 aptamers.[7] Finally, G-quadruplex structures are also receiving increasing attention because of their applications in supramolecular chemistry and nanotechnology. High-order structures, such as G-wires,[8] DNA nanodevices based on quadruplex–duplex interconversion[9] and biosensors[10] have been described in the literature. A quadruplex can be tetramolecular, bimolecular or unimolecular. Within these groups, these structures can be classified on the basis of the relative orientation of the chains (parallel or antiparallel) and the way the loops connect the different strands.[11]

Synthetic branched oligonucleotides have been used for several purposes. Initially, most of the interest in this field focused on the study of branched oligoribonucleotides as splicing intermediates of eukaryotic mRNAs.[12] Moreover, branched oligonucleotides also show high affinity for single-stranded oligonucleotides to form alternated strand triplexes.[13] Recently, branched oligonucleotides have been used as building blocks for the synthesis of new nanostructures.[14] Branched oligonucleotides carrying four G-rich strands linked through their 5’- or 3′-end have been synthesized using a clever combination of symmetric and asymmetric doubler phosphoramidites, and the resulting compounds form very stable quadruplexes.[15]

Inspired by these results, we decided to synthesize G-quadruplex branched oligonucleotides carrying modifications on one of the four strands. We used the tetrameric, G/thymidine (T)-containing parallel quadruplex [d(TG_4_T)]_4_ as a model compound to set up our synthetic strategy, since it is well-studied and its structure is well-characterized.[16–18] Moreover, this model compound has been used to assess the effects of substituting guanine for modified guanine derivatives on the stability and kinetics of quadruplex formation.[19] For example, previous work addressed the effect of 8-aminoguanine substitutions on the tetramolecular [d(TG4T)]_4_ quadruplex.[20] Thermal denaturation studies showed that an 8-aminoguanine replacement is not equally favorable at all positions, but might accelerate parallel quadruplex formation when inserted in the internal region. However, due to the tetrameric nature of the structure, it is not possible to assess the effect of a single substitution in the quadruplex.

**Scheme 1:**
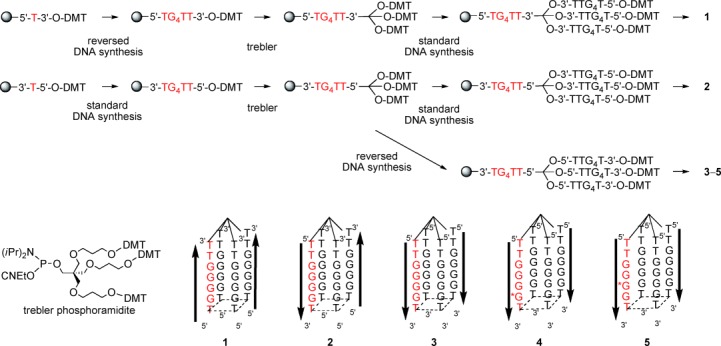
Outline of the method for the preparation of G-quadruplexes proposed in this study (see Table 2 in the Experimental Section for oligonucleotide sequences). *G denotes the position of 8-aminoguanine residues. For standard DNA synthesis, a cycle for each nucleotide addition consists of the following steps: 1) 3 % trichloroacetic acid/dichloromethane; 2) 5′-DMT-nucleoside-3′-phosphoramidite, tetrazole; 3) capping with acetic acid and *N*-methylimidazole; 4) oxidation with 0.01 m iodine solution. The same steps are applied in the reversed DNA synthesis but with the use of 3′-DMT-nucleoside-5′-phosphoramidite as monomers.

Here, we report the preparation of molecules composed of four oligonucleotide strands, whose ends are attached through a tetra-end-linker. This was achieved by first synthesizing one strand, then adding the trebler phosphoramiditethe and synthesizing the other three strands simultaneously (Scheme 1). Using the tetrameric [d(TG4T)]_4_ parallel quadruplex as a model compound, we synthesized very stable quadruplexes with modifications in only one of the strands (Scheme 1). These results are relevant for the preparation of stable quadruplexes with potential biological activity.

## Results and Discussion

### Synthesis of oligonucleotides

The synthesis of the branched oligonucleotides designed to form a monomolecular G-quadruplex was performed in an automatic DNA synthesizer (Scheme 1). The sequence of the strands was chosen based on the hexamer d(TG_4_T), which is known to form a stable parallel tetramolecular quadruplex that has been well characterized in previous studies.[16–18] In this study, an additional T was inserted to prevent steric hindrance of trebler with the nearest G-quartet. The main structural feature of these oligonucleotides is the attachment of four strand ends through a three-branched linker after the synthetic completion of one of the strands. The branched structure was incorporated into the molecule with commercially available trebler phosphoramidite (Scheme 1). By selecting standard or reversed phosphoramidites at different synthesis steps, three structures with different strand orientations were prepared (Scheme 1). The synthesis of oligonucleotide **1** started with a solid support carrying a T linked via a succinyl linker through its 5′ end (reversed-T). The first TG_4_TT strand was assembled using reversed phosphoramidites. Trebler phosphoramidite was then added followed by the assembly of the remaining three strands using standard phosphoramidites. After the assembly of the sequence, the final detritylation was not performed in order to facilitate reversed-phase HPLC purification. Ammonia deprotection generated a major eluted product containing three DMT groups. This product was collected and treated with acetic acid to obtain the desired compound, which was then characterized by MS. Oligonucleotide **1** has all four strands in the same orientation and was designed to form a monomolecular parallel G-quadruplex structure.

Oligonucleotide **2** was prepared in a similar way. This time, the first strand was assembled using a 3′-end T-linked solid support and standard phosphoramidite. Trebler phosphoramidite was then added, followed by the assembly of the remaining three strands using standard phosphoramidites. Oligonucleotide **2** had one of the strands in antiparallel polarity compared with the other three strands.

Oligonucleotide **3** was prepared using the standard 3′–5′ direction of synthesis as described for oligonucleotide **2**, however, after the addition of the trebler, the assembly of the remaining three strands was performed using reversed phosphoramidites. Thus, oligonucleotide **3** had the four strands in the same orientation as oligonucleotide **1**, but linked through the 5′-end, while in oligonucleotide **1** the strands were linked through the 3′-end. Two additional oligonucleotides carrying one single 8-aminoguanine in each position of the first strand (**4** and **5**) were prepared. Insertion of an 8-aminoguanine residue was performed with 8-amino-dG phosphoramidite protected with a dimethylaminomethylidine group.[21] The synthesis of 8-amino-guanine oligonucleotides is straightforward and requires no changes from regular procedures, with the exception of the addition of 2-mercaptoethanol to the cleavage and deprotection solutions to prevent further oxidative damage.[21] The addition of 8-aminoguanine to the parallel structure was performed to determine the effect of a single substitution in each position of the parallel quadruplex.

It is important to mention that during the synthesis of oligonucleotides **3**–**5**, the removal of the DMT group after DMT-on HPLC purification was very slow. The usual treatment (80 % acetic acid, 30 min, RT) was not sufficient to remove the three DMT groups linked to sterically hindered secondary alcohols. Instead an increased temperature was required (80 % acetic acid, 30 min, 55 °C).

### Analysis of the structure of oligonucleotides 1–3

**Figure 1:**
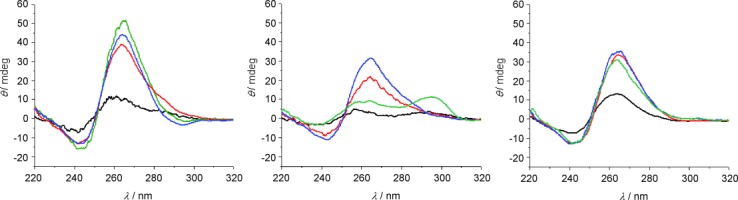
CD spectra of oligonucleotide 1 (left), 2 (middle) and 3 (right) dissolved in water (—), 5 mM KCl (—), 100 mM NaCl (—) and 100 mM NH_4_OAc (—).

CD spectra of aqueous solutions of oligonucleotide **1** show a weak positive band with a maximum around 260 nm and a negative band at 240 nm, thereby indicating the presence of residual parallel quadruplex (Figure 1). The addition of K^+^ (5 mm), Na^+^ (100 mm) and NH_4_^+^ (100 mm) enhanced the CD signal, indicating a strong stabilization of the parallel quadruplex. CD spectra of aqueous solutions of oligonucleotide **2** in water suggest that the sequence is unstructured. Upon addition of NH_4_^+^ (100 mM), two positive bands with maxima around 260 and 295 nm were enhanced. This spectrum resembles that expected for a quadruplex with three strands in one direction and one strand in an antiparallel direction (3+1 quadruplex).[22] This observation suggests that for oligonucleotide **2** in NH_4_^+^, the trebler remains on one side of the G-quadruplex and that the four strands keep the 3+1 orientation given by the synthesis. In contrast, the positive band around 295 nm disappeared and the 260 nm band increased when K^+^ (5 mm) or Na^+^ (100 mm) is added instead of NH_4_^+^. This indicates the formation of a parallel quadruplex in the presence of K^+^ and Na^+^ ions, and it is consistent with the literature on similar tetra-end-linked quadruplexes.15a Our findings show that the branching unit has enough flexibility to allow the antiparallel strand to be antiparallel in water and NH_4_^+^ solutions or to be in the parallel orientation in the presence of K^+^ and Na^+^ ions. CD spectra of oligonucleotide **3** were very similar to those of oligonucleotide **1**, thus indicating the formation of a parallel quadruplex under all conditions studied, including water, with strong stabilization by the addition of K^+^, Na^+^ and NH_4_^+^ ions.

The thermal denaturation of quadruplexes formed by oligonucleotides **1**–**3** and nonbranched [TG_4_T]_4_ was studied by UV spectroscopy (Table 1). In the presence of KCl (5 mM), all quadruplexes were stable at temperatures up to 80 °C. In the presence of Na^+^ ions (10 mM sodium cacodylate, 100 mM NaCl),[23] tetra-end linked quadruplexes **1** and **3** were stable up to 80 °C. The tetramolecular [TG_4_T]_4_ quadruplex had a melting temperature (*T*_m_) of 58 °C similar to the *T*_m_ value of oligonucleotide **2** (55 °C). This result indicates that the quadruplex formed by **2** in the presence of Na^+^ ions is the least stable. In the presence of NH_4_^+^ ions (100 mM NH_4_OAc), similar results were obtained. Only parallel tetra-end linked quadruplexes (**1** and **3**) were stable at temperatures up to 80 °C. The tetrameric [TG_4_T]_4_ quadruplexes had a *T*_m_ value of 67 °C equal to that of oligonucleotide **2**.

**Table 1:** Melting temperatures (*T*_m_)

Oligonucleotide	*T*_m_ [°C][a]			
	K^+^[b]	Na^+^[c]	NH_4_^+^[d]	H_2_O[e]
TG_4_T	>80	58	67	–
**1**	>80	>80	>80	–
**2**	>80	55	67	–
**3**	>80	>80	>80	50
**4**	>80	–	–	52
**5**	>80	–	–	>80

[a] Measured by UV spectroscopy

[b] 5 mM KCl

[c] 100 mM NaCl

[d] 100 mM NH_4_OAc

[e] Measured by CD spectroscopy.

In water after HPLC purification and desalting, could contain some residual TEAA.

**Figure 2:**
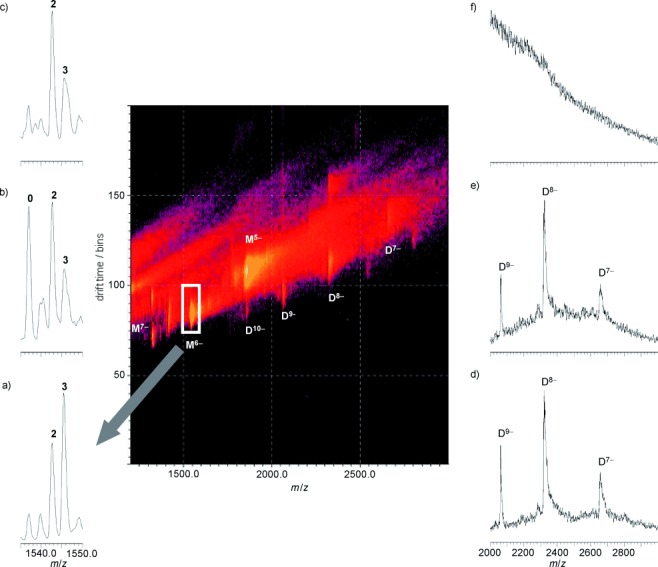
Left: ESI-MS of a quadruplex formed by oligonucleotides 1 (a), 2 (b) and 3 (c) and the distribution of the number of NH_4_^+^ ions preserved in the G-quadruplex at −6 charge state; the mass spectra were smoothed using a mean function, 2*10 channels, using MassLynx 4.0. Center: 2D ESI-MS and drift time distribution of oligonucleotide 1. Right: ESI-MS of a dimer formed by oligonucleotides 1 (d), 2 (e) and 3 (f); the mass spectra were smoothed using a mean function, 2*30 channels, using MassLynx 4.0.

ESI-MS was performed in order to study the stability of these quadruplexes in the gas phase, and hence without the boiling temperature restriction. However, this analysis is possible only with G-quadruplexes produced from ammonium solutions. In the gas phase, the number of trapped ammonium ions indicates the gas phase stability of these branched G-quadruplexes.19a Figure 2 (center) shows the bidimensional mass/mobility plots obtained for oligonucleotide **1**. The 2D plot allows the discrimination of monomeric and dimeric structures (the dimer is discussed below). To analyze the monomer, we extracted the mass spectrum corresponding to the *m*/*z* region 1535–1550. The extracted mass spectra of oligonucleotides **1**–**3** are shown in Figure 2 a–c, and the numbers indicate the number of ammonium ions preserved. Oligonucleotide **2** is the least able to preserve the inner ammonium ions in the gas phase (Figure 2), and hence is the least stable, in agreement with melting experiments in solution. Interestingly, the gas phase data indicate that **1** is more stable than **3**, although both oligonucleotides have solution *T*_m_ values above 80 °C. Overall, the relative stability of quadruplexes in the gas phase ranks **1**>**3**>**2**.

**Figure 3:**
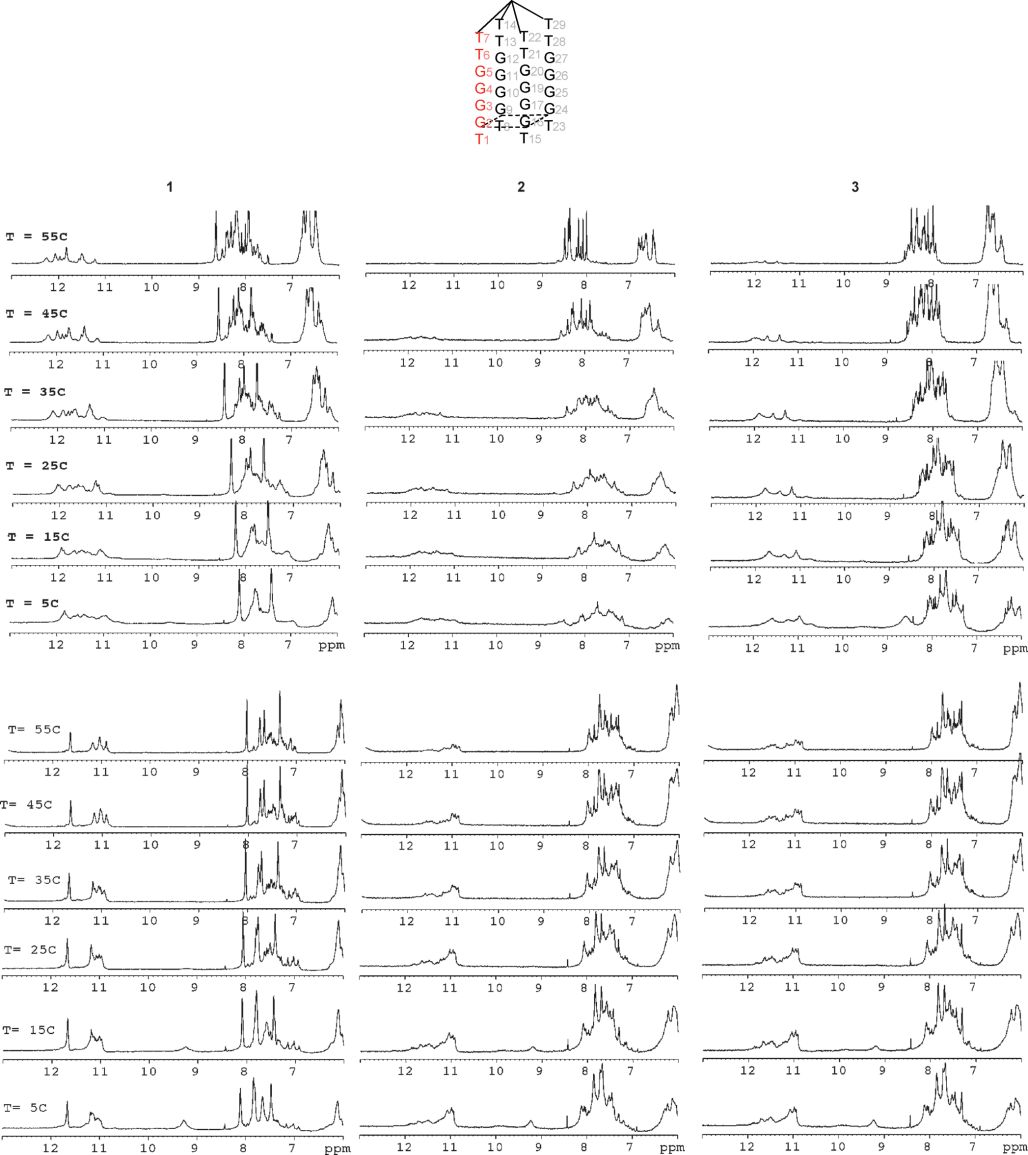
Top: General scheme of a tetra-end-linked quadruplex showing the numeration of the residues as mentioned in the text. Bottom: exchangeable proton region of ^1^H NMR spectra at different temperatures of oligonucleotides 1, 2 and 3 in 10 mM sodium phosphate buffer (upper rows) or 10 mM potassium phosphate buffer (lower rows).

The imino proton region of the NMR spectra of oligonucleotides **1**–**3** confirms the formation of a quadruplex structure (Figure 3). NMR spectra were acquired in Na^+^ and K^+^ buffers. Under both conditions, the spectra of the three oligonucleotides exhibited imino signals at *δ* values in the range of 10.4–11.5 ppm, characteristic of imino protons involved in the Hoogsteen N1H–O6 hydrogen bonds of G-quartets. These imino signals were observed at high temperatures, indicating that the three quadruplexes were very stable, being more stable under K^+^ than Na^+^ conditions. The relative stability between the three oligonucleotides was in agreement with UV-melting experiments (**1**> **3**> **2**). At low temperatures, NMR signals were broad, suggesting the presence of more than one species in equilibrium. In quadruplex **1**, signals became sharper upon temperature increase. This effect was more pronounced in K^+^ buffer and is probably due to the dissociation of multimeric species. To determine the oligomerization state of the samples, we performed native gel electrophoreses (see below).

**Figure 4:**
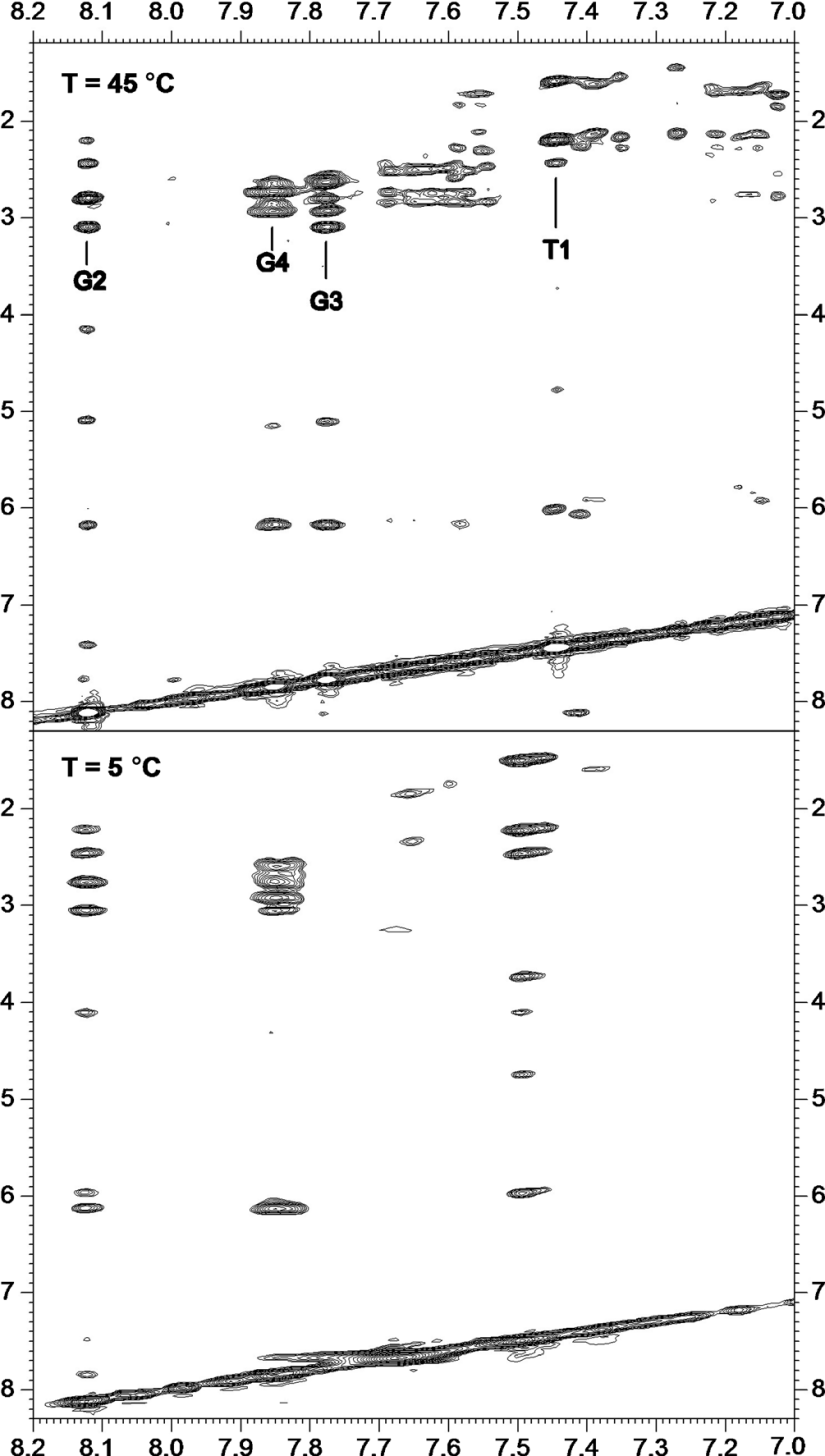
Fragments of NOESY spectra (250 ms mixing time) of oligonucleotide 1 at 5 °C (bottom) and 45 °C (top) in 10 mM potassium phosphate (pH 7).

NMR spectra of **1** in K^+^ buffer conditions were of sufficient quality to acquire 2D experiments. On the basis of NOESY and TOCSY spectra, four spin systems with relatively sharp signals were clearly identified, and they were sequentially assigned to residues 1–4. NOE cross-peak patterns for these residues indicate that the four chains are equivalent and the guanines adopt an *anti*-conformation. The remaining nucleotides (G5, T6 and T7) presented a broad signal and their NOE cross-peaks were almost invisible at low temperatures (Figure 4). At higher temperatures, these residues exhibited multiple cross-peaks in the NOESY spectra (Figure 4), suggesting the presence of several conformers in this region of the molecule. The presence of these conformers is possibly related to a conformational heterogeneity that affects the nucleotides near the linker. This is consistent with the number of signals and their relative intensities observed in the 1D spectra at this temperature (Figure 3).

Methyl-imino cross-peaks in the exchangeable proton region of the NOESY experiment allowed for the assignment of H1 of G2 (Figure S2 in the Supporting Information). Also an imino–imino sequential cross-peak between H1G2 and H1G3 was clearly observed. Other exchangeable protons corresponding to G4 and G5 were also observed, but could not be specifically assigned. The number of exchangeable proton signals and their cross-peak pattern indicated the formation of four guanine tetrads.

In summary, the NMR experiments indicate that at high temperature oligonucleotide **1** forms a symmetrical parallel quadruplex, most probably monomeric, where the four chains are equivalent and all guanines adopt an *anti*-conformation. Although oligonucleotide **1** exhibits a single global fold, some conformational heterogeneity occurs in residues close to the linker. This is probably due to steric constraints provoked by the linker, which impede the interconversion between different conformers. At low temperatures, NMR signals are broader suggesting an equilibrium with other species of higher molecular weight.

### Presence of dimeric quadruplex structures

Native polyacrylamide gel electrophoresis (PAGE) has been widely used to detect oligomers and aggregates. Electrophoretic analysis was carried out using the tetramolecular quadruplex [d(TG_4_T)]_4_ as a reference. First, native PAGE was performed to assess the oligomerization state of the quadruplexes studied by NMR (oligonucleotides **1**–**3**). In all cases oligonucleotides resulted in two major bands suggesting that they form not only monomeric species but also dimeric structures (Figure S1 in the Supporting Information). Also, in all cases the band corresponding to the monomer was the major band (60–70 %). ESI-MS showed that branched oligonucleotide sequences (**1**–**3**) to some extent form dimers in ammonium acetate (100 mM). The ESI-MS spectra recorded for three sequences are shown in Figure 2 d–f. Quadruplex **3** had a lower signal-to-noise ratio as a result of the presence of residual salts, but in all three cases, the presence of a dimeric quadruplex as a minor component was confirmed by mass spectrometry.

**Figure 5:**
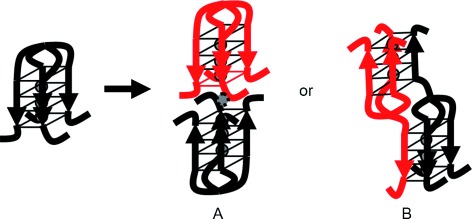
Hypothetical dimerization of the quadruplex-forming structures.

We propose two hypothetical dimeric structures for the dimer formed by these branched oligonucleotides. The association of two molecules allows the formation of two parallel quadruplexes, each containing one strand belonging to the adjacent molecule (e.g., interlocked structure **B** shown in Figure 5). The second model proposed is based on previous observations reported in the literature (structure **A** in Figure 5). Crystallographic studies of d(TG_4_T) quadruplexes performed by Cáceres et al.[24] revealed the stacking of T tetrads between neighboring quadruplexes packed in a head-to-head fashion. Recent NMR studies on UG_4_U revealed the existence of a dimeric quadruplex structure in the presence of K^+^ and NH_4_^+^ but not Na^+^ ions.[25] ESI-MS studies on telomeric sequences performed by Collie et al[26] revealed that telomeric RNA form higher-order dimeric assemblies, initiated by cation-mediated stacking of two parallel G-quadruplex subunits.

This dimer, consistent with two G-quadruplex subunits, each with three NH_4_^+^ ions, plus one NH_4_^+^ ion stacked between the two subunits was observed by ESI-MS experiments. This result suggests a structural model for the dimer involving the cation-mediated stacking of G-quadruplex subunits (structure **A** in Figure 5).

### Analysis of the stability of oligonucleotides carrying 8-aminoguanine

CD studies and thermal denaturation were performed in order to study the effect of 8-aminoguanine substitution on the stability of the quadruplex. As described above, in the presence of K^+^ ions (5 mM KCl), all quadruplexes were stable up to 80 °C as no changes in the UV spectra were observed. For this reason, oligonucleotides **3**–**5** were dissolved in water and CD thermal denaturation was performed (see the Supporting Information). Under these conditions, the samples contained some residual triethylammonium acetate (TEAA) from HPLC purification. Even in the absence of K^+^ and Na^+^ ions, the CD spectra of oligonucleotides **3**–**5** showed the presence of a parallel quadruplex structures: a positive band with a maximum at 260 nm and a negative band with a minimum at 240 nm. In the absence of K^+^ and Na^+^ ions, unmodified oligonucleotide **3** had a *T*_m_ value of 50 °C (Table 1). The introduction of an 8-aminoguanine residue in the external position of the quadruplex (oligonucleotide **4**) produces a quadruplex with similar stability (52 °C). In contrast, the substitution of a single G by 8-aminoguanine in the internal quartet (oligonucleotide **5**) induced the formation of a very stable quadruplex, as only a partial melting was observed at 80 °C.

## Conclusion

Here, we have used the trebler branching unit to synthesize molecules containing four G-rich DNA strands linked by one of the ends. These molecules form very stable parallel G-quadruplex structures even in the absence of stabilizing cations. NMR experiments confirmed that oligonucleotide **1** forms a symmetrical parallel quadruplex, where the four chains are equivalent and all guanines adopt an *anti*-conformation. The methodology described here allowed the synthesis of quadruplexes with single modifications in one of the strands. Oligonucleotides with 8-aminoguanine substitutions provide more stable structures when the replacement is carried out in the internal region of the quadruplex. This system has also allowed the synthesis of quadruplexes with a single antiparallel strand. In this case, all-parallel or 3+1 structures are observed depending on the cation present in the solution. Surprisingly, in addition to the monomolecular G-quadruplex, bimolecular structures are also observed. These bimolecular species were not detected in previous studies on branched G-quadruplexes[15] and could be interesting for biomedical applications, as it has been observed that in HIV-binding aptamers these species are relevant for the HIV inhibitory properties.[7] The method described here provides an excellent platform to obtain defined and stable G-quadruplex structures that could provide advantages to the conventional tetramolecular quadruplex in the development of applications, such as convenient functionalization as well as thermodynamic and biological stability.

## Experimental Section

### Synthesis of oligonucleotides

The oligonucleotide sequences used in this study (Table 2) were prepared on either a 0.2 μmol scale for oligonucleotides **3**–**5** or 1 μmol scale for oligonucleotides **1**, **2** using commercially available phosphoramidites and polystyrene (LV200) or controlled pore glass (CPG) solid supports. The trebler phosphoramidite was obtained from Glen Research (Sterling, VA, USA). 2′-Deoxyguanosine was protected with an isobutyryl group and 2′-deoxycytidine and 2′-deoxyadenosine with a benzoyl group. Reversed phosphoramidites were from Link Technologies (Lanarkshire, UK). The phosphoramidite of 8-amino-2′-deoxyguanosine was protected with two dimethylaminomethylidene groups obtained from Berry Associates (Dexter, MI, USA). Oligonucleotides were purified by HPLC using a Nucleosil C_18_ column (120–10, 250×4 mm, Macherey-Nagel, Düren, Germany). Elution was performed with a mixture of solution A (5 % CH_3_CN in 0.1 M aq triethylammonium acetate (TEAA)) and solution B (70 % CH_3_CN in 0.1 M aq TEAA) at a flow rate of 3 mL min^−1^ in 20 min (15–80 % B, DMT off conditions).

**Table 2:** Oligonucleotide sequences used in this study

Quadruplex	Sequence[a]
**1**	5′-TGGGGTT-3′-TB-[3′-TTGGGGT-5′]3
**2**	3′-TGGGGTT-5′-TB-[3′-TTGGGGT-5′]3
**3**	3′-TGGGGTT-5′-TB-[5′-TTGGGGT-3′]3
**4**	3′-TG^*^GGGTT-5′-TB-[5′-TTGGGGT-3′]3
**5**	3′-TGG^*^GGTT-5′-TB-[5′-TTGGGGT-3′]3

[a] G^*^: 8-amino-G nucleotides. TB: trebler linker [–O–phosphate–CH_2_–C(CH_2_OCH_2_CH_2_CH_2_O–phosphate–)_3_].

The first part of oligonucleotide **1** was prepared using reversed 5′-phosphoramidites. The coupling time for reversed 5′-phosphoramidites was increased to 3 min, while that for the trebler phosphoramidite was increased to 15 min. After trebler phosphoramidite incorporation, the three remaining strands were assembled using standard 3′-phosphoramidites. Oligonucleotide **2** was prepared using standard 3′-phosphoramidites for the entire synthesis. The first parts of oligonucleotides **3**–**5** were prepared using standard 3′-phosphoramidites. 8-Amino-2′-deoxyguanosine phosphoramidite was used to introduce the 8-aminoguanine residue at the desired positions. The coupling time for 8-aminoguanine phosphoramidite was increased to 5 min. After the incorporation of the trebler phosphoramidite the three remaining strands were assembled using reversed 5′-phosphoramidites. Generally, solid supports were treated with concd aq NH_3_ (1 ml) at 55 °C overnight. Supports carrying oligonucleotides **4** and **5** were treated with concd NH_3_ (1 ml) containing 2-mercaptoethanol (0.1 m) at 55 °C for 24 h. After filtration, the solid supports were washed with H_2_O (3 ml), and the combined solutions were evaporated in vacuo to dryness. The residues were dissolved in H_2_O, and the oligonucleotides were purified by HPLC. The purified products were treated with 80 % aq AcOH at RT for 30 min, except for oligonucleotides **3**–**5**, which were treated at 55 °C for 30 min because 3′-dimethoxytrityl (3′-DMT) is more resistant to detritylation. AcOH was extracted with Et_2_O (3×5 ml), and the resulting compounds were desalted using a NAP-10 column and analyzed using a 4800 Plus MALDI TOF/TOF Analyzer (AB Sciex, Framingham, MA, USA). At 260 nm, yields were around 2 OD units for the 0.2 μmol scale and 15 OD units for the 1 μmol scale.

Oligonucleotide **1**: [*M*−H]^−^ calcd: 9227.5, found: 9227; oligonucleotide **2**: [*M*−H]^−^ calcd: 9227.5, found 9227; oligonucleotide **3**: [*M*−H]^−^ calcd: 9227.5, found: 9227; oligonucleotide **4**: [*M*−H]^−^ calcd: 9242.5, found 9236; oligonucleotide **5**: [*M*−H]^−^ calcd: 9242.5, found 9233.

### Thermal denaturation experiments

**UV spectroscopy**: The thermal melting curves for oligonucleotides **1**–**5** and d(TG_4_T)_4_ were performed following the UV-absorption change at 240, 260 and 295 nm in a temperature range of 20–95 °C with a linear temperature ramp of 0.5°C min^−1^ on a JASCO V-650 spectrophotometer equipped with a Peltier temperature control. The measurements were conducted in 5 mM KCl, 100 mM NaCl, 100 mM NH_4_OAc or Milli-Q H_2_O. Oligonucleotides (5 μm) were annealed by heating to 95 °C and slowly cooling to RT before measurement.

**CD spectroscopy**: CD spectra were obtained from a spectropolarimeter (Jasco) equipped with a Peltier temperature control. CD spectra were registered between 220 and 320 nm in either Milli-Q H_2_O, 5 mM KCl, 100 mM NaCl or 100 mM NH_4_OAc. CD thermal denaturation experiments were performed in the temperature range of 10–90 °C using a heating rate of 0.5 °C min^−1^ and monitoring the CD values at 260 nm. Oligonucleotides (5 μm) were annealed by heating the samples to 95 °C and slowly cooling to RT before recording CD spectra and melting profiles.

### NMR spectroscopy

Samples for NMR measurements were dissolved in H_2_O/D_2_O (9:1, 200 μL) containing either NaH_2_PO_4_/Na_2_HPO_4_ (10 mm) or KH_2_PO_4_/K_2_HPO_4_ (10 mM). Oligonucleotide concentrations were ∼300 μM for oligonucleotide **1**, and ∼150 μM for **2** and **3**. ^1^H NMR spectra were collected at temperatures ranging from 5 to 55 °C on a AV-600 spectrometer (Bruker, Billerica, MA, USA) equipped with a cryoprobe. Water suppression was achieved by including a WATERGATE module in the pulse sequence prior to spectra acquisition.

### ESI-MS and Ion mobility spectrometry (IMS)

The ESI-MS experiments described were recorded in ion mobility mode using a SYNAPT G2 HDMS (Waters, Manchester, UK). The capillary voltage was set to −2.2 kV; cone voltage=30 V; extraction cone=4 V; source pressure=3.15 mbar; source and desolvation temperatures=40 °C; trap and transfer voltages=4 V. The helium cell is supplied with helium at 180 mL min^−1^, and the ion mobility cell is supplied with N_2_ to reach a pressure of 3.88 mbar in the IMS cell (instrument pirani reading). The wave height was 40 V and the wave speed was 1000 m s^−1^. The bias voltage for ion introduction into the IMS cell was 35 V. Both instruments were calibrated in collision cross section with oligonucleotides, as described previously.[27] Oligonucleotides **1**, **2** and **3** (200 μm) were folded in NH_4_OAc buffer (100 mm) and injected at a final strand concentration of 5 μM and at a rate of 140.0 μL h^−1^ at RT.

## Supplementary material

Detailed facts of importance to specialist readers are published as ”Supporting Information”. Such documents are peer-reviewed, but not copy-edited or typeset. They are made available as submitted by the authors.
