# Supplementary material for: Synthesis and Structural Characterization of Stable Branched DNA G-Quadruplexes Using the Trebler Phosphoramidite
Source: ChemistryOpen. 2012 Apr;1(2):106–14. doi: 10.1002/open.201200009 (PMC3922461; doi:10.1002/open.201200009)
Supplement: Supplementary file 1 [file open0001-0106-SD1.pdf]

ChemistryOPEN

Supporting Information

© Copyright Wiley-VCH Verlag GmbH & Co. KGaA, 69451 Weinheim, 2012

Synthesis and Structural Characterization of Stable Branched DNA G-Quadruplexes Using the Trebler Phosphoramidite

Rubén Ferreira,[a, b] Margarita Alvira,[a, b] Anna Aviñó,[a, b] Irene Gómez-Pinto,[c] Carlos González,[c] Valérie Gabelica,[d] and Ramon Eritja*[a, b]

open_201200009_sm_miscellaneous_information.pdf

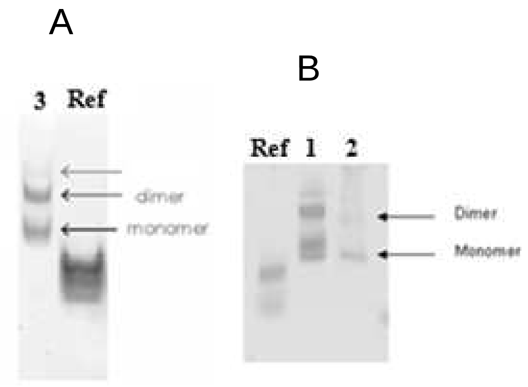

Figure S1. Native gel electrophoresis of A) oligonucleotide **3** and B) oligonucleotides **1** and **2**. In both cases $[d(TGGGGT)]_4$ was used as reference (Ref) for comparison of relative migrations.

Gel electrophoresis experiments were performed on 15% native bis/acrylamide gels at 4ºC. The electrophoresis was run at this temperature at 200 V in a SE-600 Hoefer Scientific apparatus using TAE Mg^{2+} (40 mM Tris, 2mM EDTA, 20 mM AcOH, and 12.5 mM magnesium acetate) as running buffer. The final concentration of oligonucleotides **1**, **2**, **3** and $[d(TGGGGT)]_4$ sample solutions were 25 µM. The samples were annealed in 5 mM K^+ buffer and then dissolved in loading buffer containing 50% glycerol in TAE Mg^{2+} buffer to obtain the final concentration. After the electrophoresis, oligonucleotides were stained by STAINS-ALL (Sigma).

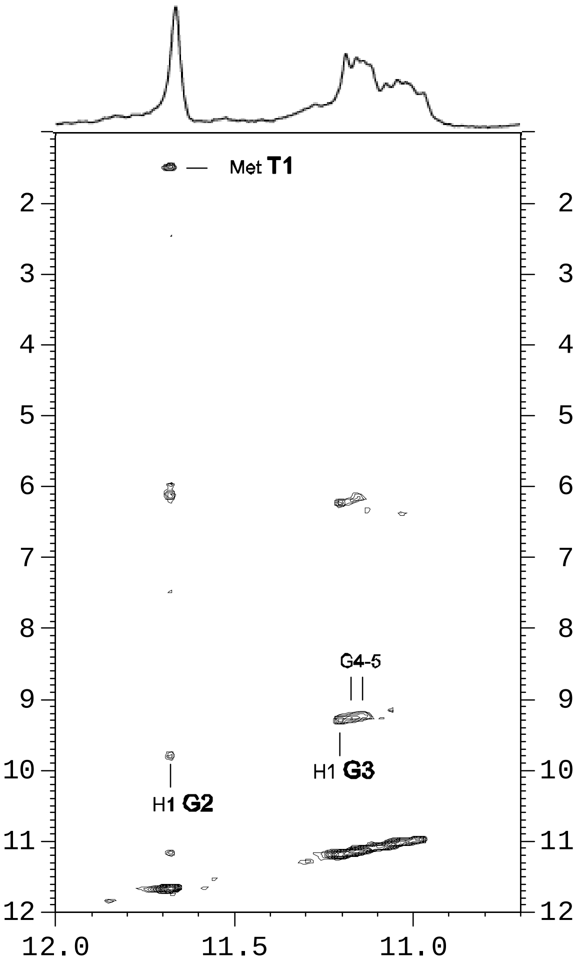

Figure S2. Imino proton region of the NOESY (100ms mixing time) spectra of oligonucleotide **1** in H_2O (5mM K+ concentration, pH 7, T= 5 ºC). The corresponding region of 1D spectrum is shown on the top.

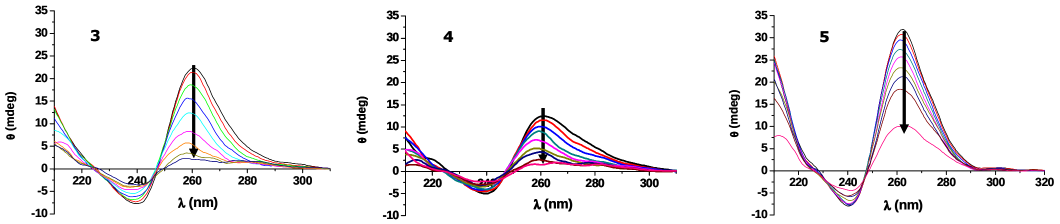

Figure S3. CD spectra of sequences **3-5** registered at different temperatures in the range 10-90 ºC. The spectra show a decrease of the CD signal upon heating.

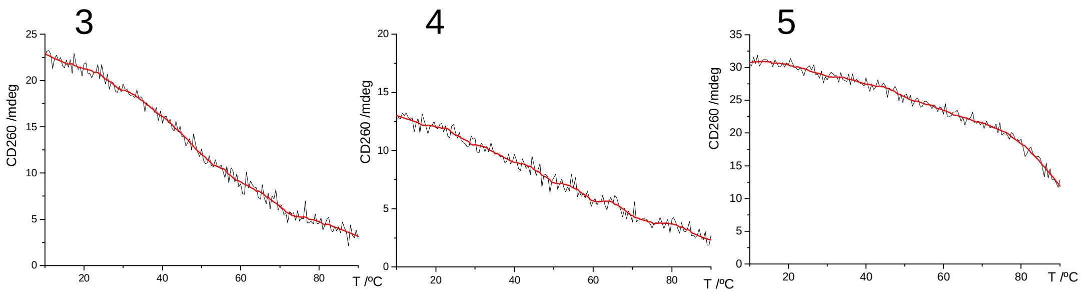

Figure S4. Melting profile of sequences **3-5** monitoring the CD values (mdeg) at 260 nm in the range 10-90 ºC using a heating rate of 0.5 ºC/min.
